# Supplementary material for: Locus coeruleus-CA1 projections are involved in chronic depressive stress-induced hippocampal vulnerability to transient global ischaemia
Source: Nat Commun. 2019 Jul 3;10:2942. doi: 10.1038/s41467-019-10795-9 (PMC6610150; doi:10.1038/s41467-019-10795-9)
Supplement: Supplementary file 1 — Supplementary Information [file 41467_2019_10795_MOESM1_ESM.pdf]

SUPPLEMENTARY INFORMATION

Locus coeruleus-CA1 projections are involved in chronic depressive stress-induced hippocampal vulnerability to transient global ischaemia

Zhang et al.

Supplementary Figure 1

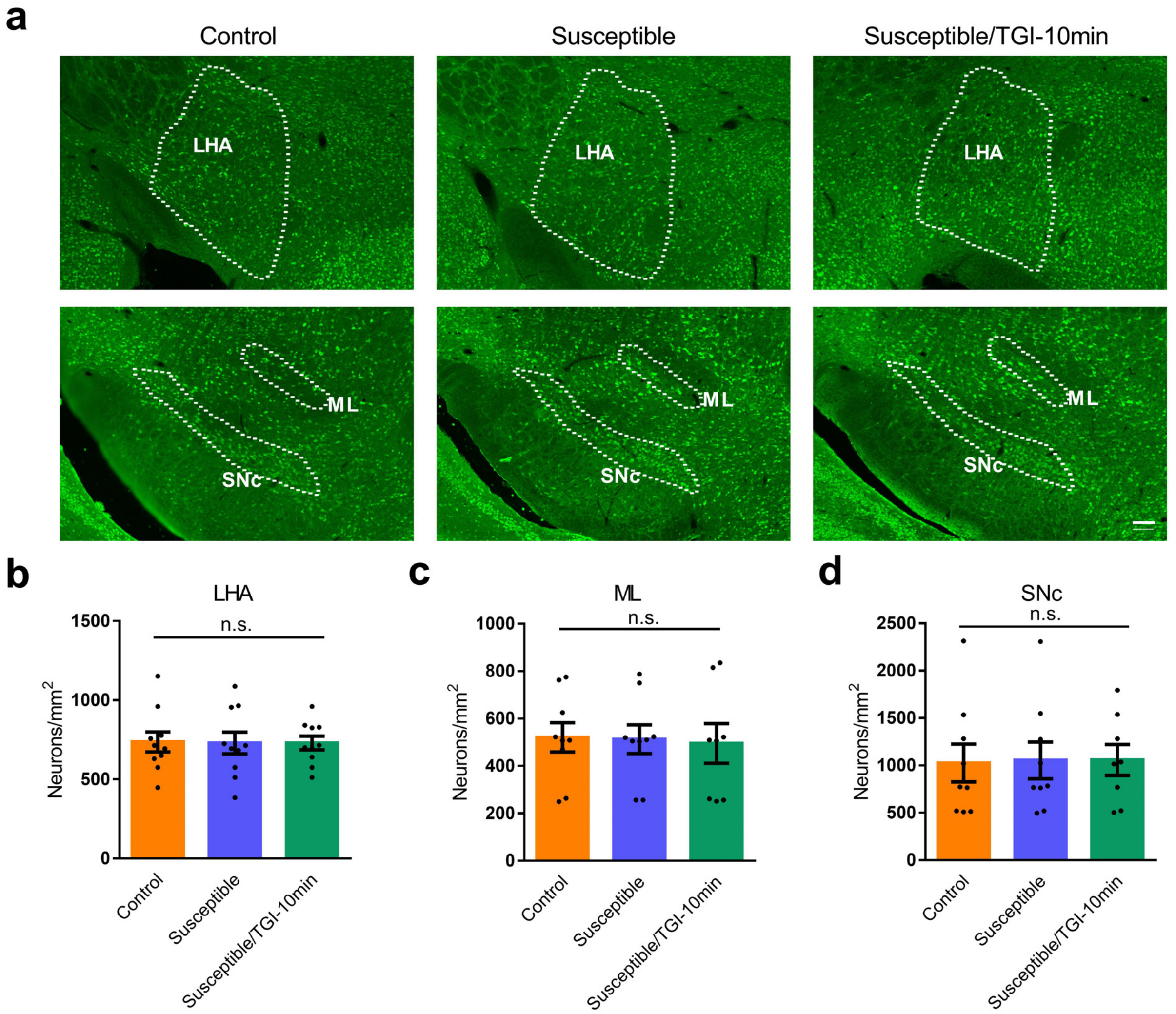

Supplementary Figure 1. The neuronal counting was not changed in LHA, ML, and SNc regions in the susceptible group with TIA-10min treatment. (a) Representative NeuN fluorescent immunostaining of LHA, ML, and SNc area in the control, susceptible, and susceptible/TGI groups. NeuN-immunoreactivity was showed as green and the nuclei were stained with DAPI for blue. Scaling bar=125μm. **(b)** Neuronal survival in LHA region. One-way ANOVA, P=0.9961. Control(n=10), susceptible(n=10), susceptible/TGI-10min(n=10). **(c)** Neuronal survival in ML region. One-way ANOVA, P = 0.9649, control (n = 9), susceptible (n=9), susceptible/TGI-10min(n=8). **(d)** Neuronal survival in SNc region. One-way ANOVA, P = 0.9913, control (n = 9), susceptible (n=9), susceptible/TGI-10min(n=8). Data are presented as means ± s.e.m. and ns indicates no significance.

Supplementary Figure 2

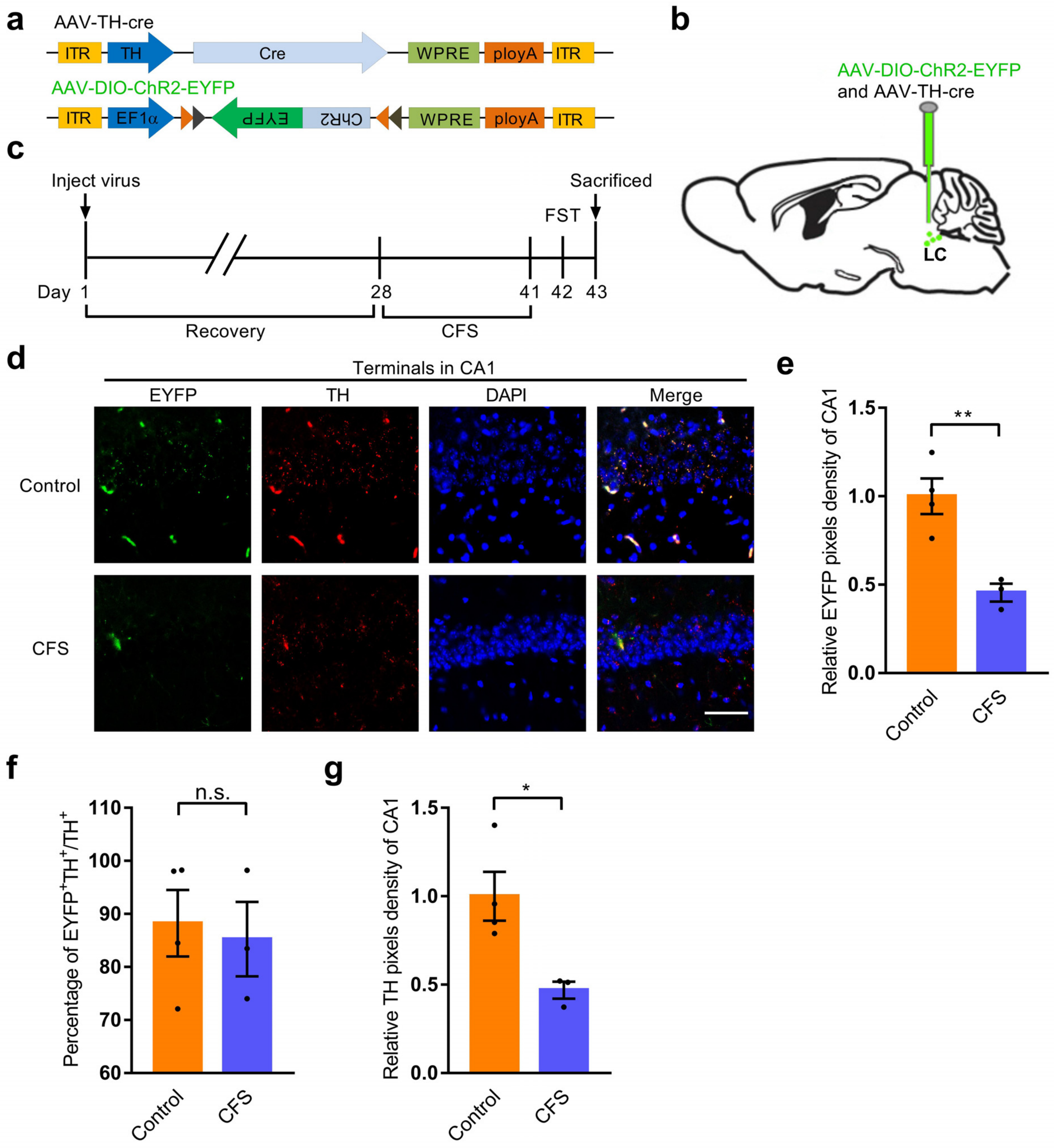

Supplementary Figure 2. *Th*:LC-CA1 projections was reduced in CFS mice. (a) Schematic representation of the construct of the AAV-DIO-ChR2-EYFP and AAV-TH-Cre virus. **(b)** Schematic of injecting mixture of viruses into LC area. **(c)** Strategy for labelling the direct outputs of LC-TH^+ neurons in control and CFS mice. **(d)** Representative immunofluorescent staining shows the EYFP (green), anti-TH (red), and DAPI (blue) of CA1 area. Scaling bar=250μm. **(e)** Quantitation of pixel density of TH:LC axon projections (EYFP positive) in CA1 region in control and CFS mice. Unpaired two-tailed Student's t-test, P = 0.0076**. **(f)** Percentage of $EYFP^+TH^+$ double positive cells in the whole TH^+ cells in CA1 area in control and CFS mice. Unpaired two-tailed Student's t-test, P = 0.7646. **(g)** Quantitation of pixel density of TH^+ axon projections in CA1 region in control and CFS mice. Unpaired two-tailed Student's t-test, P = 0.0253*. All data are presented as means ± s.e.m. Control(n=4), CFS(n=3), ns: no significant.

Supplementary Figure 3

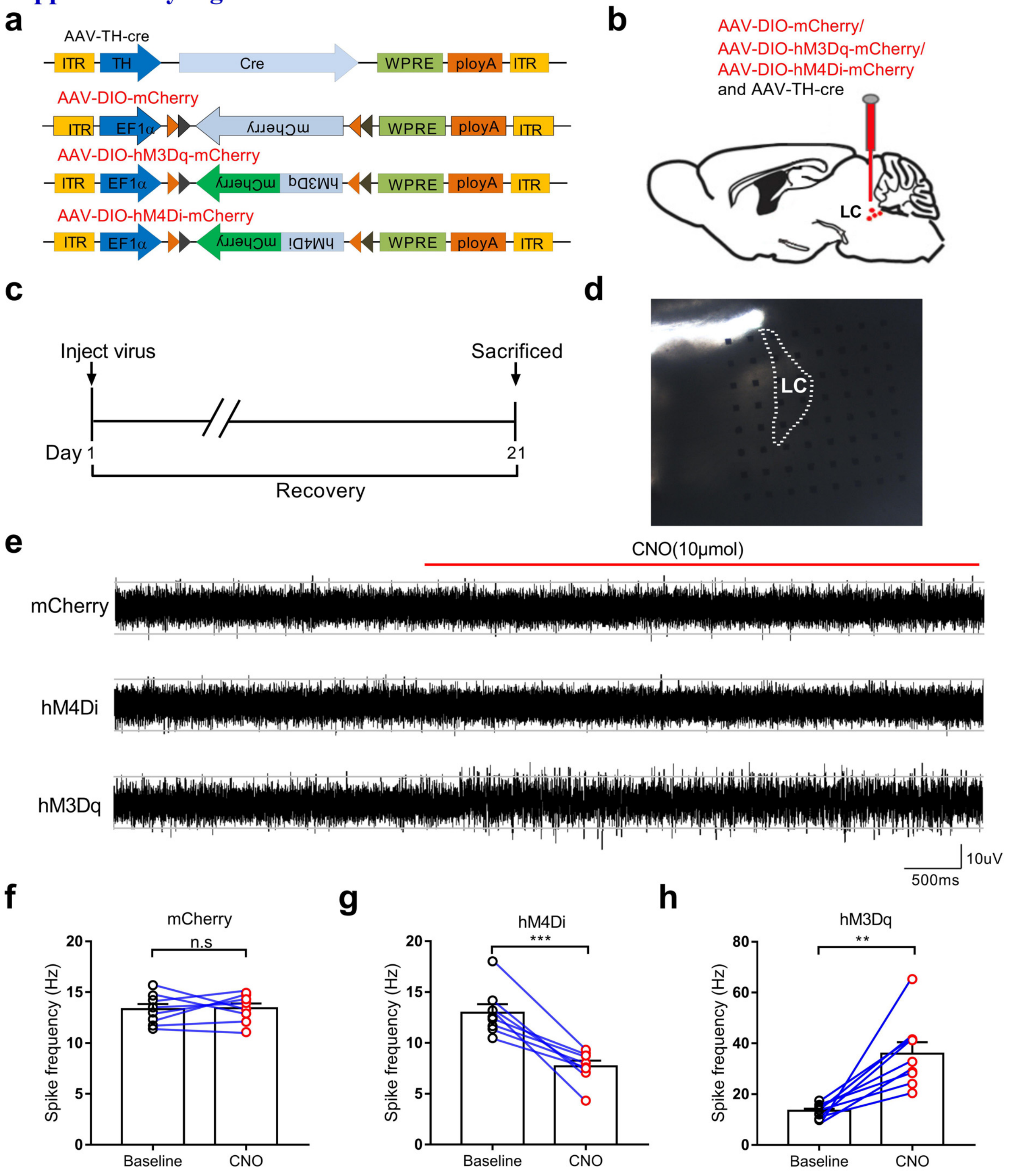

Supplementary Figure 3. Electrophysiological recording of LC neurons after exogenous expression DREADDs with CNO bath application. (a) Schematic representation of the AAV-DIO-mCherry, AAV-DIO-hM4Di-mCherry, AAV-DIO-hM3Dq-mCherry and AAV-TH-Cre virus. **(b)** Diagrammatic drawing of injecting mixture of viruses into LC region. **(c)** Timeline of experiments. **(d)** Example of cornel slices with 8 × 8 MEA placed over the LC area. **(e)** Representative trace of spontaneous activity in LC region. **(f)** Spontaneous activity of LC region before and after CNO bath application in mCherry-only group (n = 8 slices in 7 mice, *P* = 0.8598). **(g)** Aligned dot plot of electrophysiology recording in LC-including brain slices of hM4Di-mCherry expression (n = 8 slices in 6 mice, *P* = 0.0005***). **(h)** Histogram depicting the frequency of LC cells in hM3Dq-mCherry expression sections (n = 9 slices in 7 mice, *P* = 0.0014**). Data are represented as means ± s.e.m. Statistical analysis of (f-h) was performed by two-tailed paired Student's t test. ns: no significant.

Supplementary Figure 4

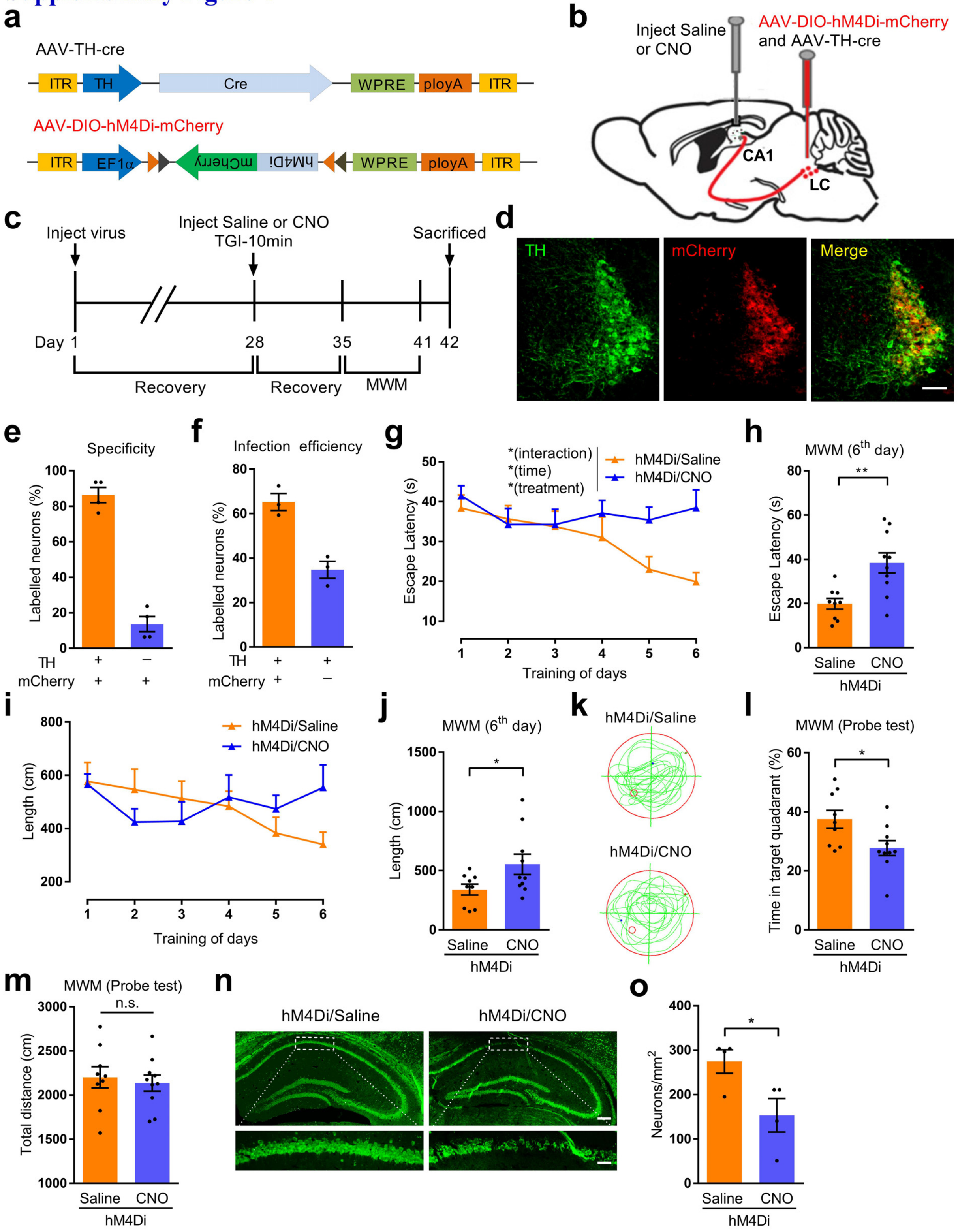

Supplementary Figure 4. Chemogenetic suppression of *TH*:LC-CA1 circuit by hM4Di/CNO reduced the MWM performance and CA1 neuronal survival in TGI-10min-treated mice. (a) Schematic of the AAV-DIO-hM4Di-mCherry and AAV-TH-Cre virus. **(b)** Diagrammatic representation of injecting mixture viruses into LC area. **(c)** Workflow of the experiments conducted. **(d)** Representative immunofluorescent staining shows targeting of hM4Di-mCherry to LC-TH^{+} neurons (green, TH, red, hM4Di-mCherry, scale bars=100μm). **(e)** Quantification of the specificity of Cre-inducible AAV in the LC of wild-type mice (4 sections in 4 mice). **(f)**

Quantification of the expression rate of the Cre-inducible AAV in the LC of normal individuals (3 slices in 3 mice). **(g)** The escape latency of the MWM test in hM4Di/Saline and hM4Di/CNO group. hM4Di/Saline(n=9) and hM4Di/CNO group(n=10). RM two-way ANOVA, interaction: P=0.0205*, time: P=0.0111*, treatment: P=0.0498*. **(h)** The latency to reach a hidden platform during the spatial learning trail on day 6. Unpaired two-tailed Student's t-test, P=0.0027*. **(i)** The swimming length of the training session on day 6. RM two-way ANOVA, interaction: P=0.1045, time factor: P=0.2859, treatment factor: P=0.6496). **(j)** Histogram depicting the swimming length to reach platform during the spatial learning test on day 6. Unpaired two-tailed Student's t-test, P=0.049*. **(k)** Representative tracing of hM4Di/Saline and hM4Di/CNO mice in probe trail on day 7. **(l)** Percentage of time spent in target quadrant during the probe test on day 7. Unpaired two-tailed Student's t-test, P=0.0227*. **(m)** Total swimming distance in the probe test on day 7. Unpaired two-tailed Student's t-test, P=0.6678. **(n)** Representative NeuN staining images show hippocampal neurons in CA1 region (green, NeuN, scale bars=100μm). **(o)** Quantitative assessment of CA1 pyramidal neuronal survival in hM4Di/Saline mice (n=4) and hM4Di/CNO group (n=4). Unpaired two-tailed Student's t-test (P=0.0392*). Data are presented as the means ± s.e.m.

Supplementary Figure 5

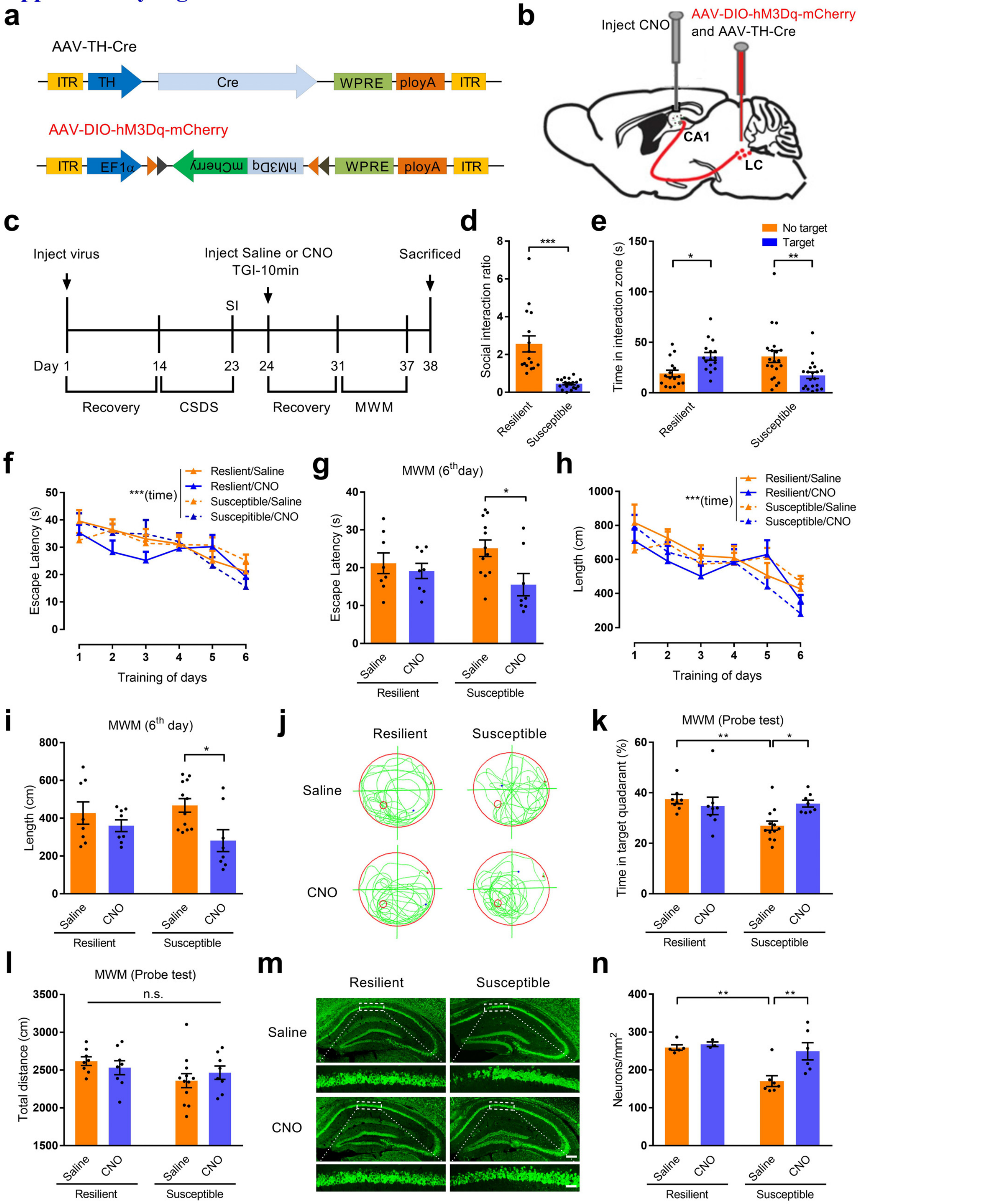

Supplementary Figure 5. DREADDs-activation of the Th:LC-CA1 circuit rescued the TGI-induced spatial memory impairment and CA1 neuronal death in CSDS susceptible mice. (a) The construct of the AAV-DIO-hM3Dq-mCherry and AAV-TH-Cre virus. **(b)** The mixture of AAV-DIO-hM3Dq-mCherry and AAV-TH-Cre

viruses was bilaterally injected into the LC region of wild-type mice to selectively target the hM3Dq-mCherry in TH-positive neurons. **(c)** Experimental workflow. **(d)** The distribution of the social interaction ratio for resilient and susceptible mice in CSDS experiments. Resilient (n=16), susceptible(n=20). Unpaired two-tailed Student's t-test, $P < 0.0001$***. **(e)** The time in the social interaction zone was recorded in resilient and susceptible groups. Two-way ANOVA, interaction: P=0.0001***, Resilient vs. Susceptible: P=0.8325, No target vs. Target: P = 0.85, post hoc Dunnett's test, Resilient/No target vs. Resilient/Target, P=0.0227*, Susceptible/No target vs. Susceptible/Target, P = 0.0043**. **(f)** Spatial learning curves in the MWM test displaying escape latency to reach the platform. Resilient/saline (n=8), resilient/CNO (n=8), susceptible/saline (n=12), susceptible/CNO (n=8). RM two-way ANOVA, interaction: P=0.2477, time: P<0.0001***, treatment: P=0.5459. **(g)** The escape latency to reach a hidden platform during the training tests on day 6. Two-way ANOVA, interaction: P=0.1414, Resilient vs. Susceptible: P=0.9455, Saline vs. CNO: P=0.0273*, post hoc Dunnett's test, Susceptible/Saline vs. Susceptible/CNO, P=0.0213*, Resilient/Saline vs. Susceptible/Saline, P=0.5155. **(h)** Total distance travelled to find the hidden platform in the MWM test during the 6-day training phase. RM two-way ANOVA, interaction: P=0.4053, time factor: P<0.0001***, treatment: P=0.4683). **(i)** The swimming length to reach a hidden platform on day 6 during the training phase. Two-way ANOVA, interaction: P=0.2083, Resilient vs. Susceptible: P=0.6821, Saline vs. CNO: P=0.0107*, post hoc Dunnett's test, Susceptible/Saline vs. Susceptible/CNO, P=0.0155*, Resilient/Saline vs. Susceptible/Saline, P=0.8556). **(j)** Representative swimming traces of mice in the probe trail on day 7. **(k)** Percentage of the time spent in the target quadrant in the probe test on day 7. Two-way ANOVA, interaction: P=0.016*, Resilient vs. Susceptible: P=0.04*, Saline vs. CNO: P=0.1899, post hoc Dunnett's test, Susceptible/Saline vs. Susceptible/CNO, P=0.0190*, Resilient/Saline vs. Susceptible/Saline, P=0.0042**). **(l)** The total distance of swimming of probe test on day 7. Two-way ANOVA, interaction: P=0.2895, Resilient vs. Susceptible: P=0.0782, Saline vs. CNO: P = 0.9137. **(m)** Representative immunofluorescence shows the cell survival of CA1 neuronal death (green, NeuN, scale bars, upper for 250μm and lower for 50μm respectively). **(n)** Quantification of the number of $NeuN^+$ neurons in hippocampus CA1. Resilient/Saline(n=5), Resilient/CNO (n=3), Susceptible/Saline (n=7), Susceptible/CNO (n=6). Two-way ANOVA, interaction: P=0.0646, Resilient vs. Susceptible: P=0.0078**, Saline vs. CNO: P=0.025*, post hoc Dunnett's test, Susceptible/Saline vs. Susceptible/CNO, P=0.0053**, Resilient/Saline vs. Susceptible/Saline, P=0.0031. Data are presented as the means ± s.e.m.

SUPPLEMENTARY METHODS

Electrophysiological recording

For obtaining slices containing LC area, mice were anesthetized with chloral hydrate (350 mg/kg) and xylazine (10 mg/kg) and perfused transcardially with an ice-cold cutting solution containing sucrose 213 mM, KCl 2.5 mM, NaH_2PO_4 1.25 mM, $MgSO_4$ 10 mM, $CaCl_2$ 0.5 mM, $NaHCO_3$ 26 mM, and glucose 11 mM (300–305 mOsm), equilibrated with 95% O_2 and 5% CO_2, pH 7.4. Mice were then rapidly decapitated and coronal 300 μM slices containing LC area were cut with a vibratome (VT1000S, Leica, Germany) at slicing speed of 2.00 and a blade vibration frequency of 8. Slices were incubated for at least 1 h in 34 °C artificial cerebral spinal fluid containing NaCl 126 mM, KCl 2.5 mM, NaH_2PO_4 1.25 mM, $MgCl_2$ 2 mM, $CaCl_2$ 2 mM, $NaHCO_3$ 26 mM, and glucose 10 mM (300–305 mOsm) (saturated with 95% O_2 and 5% CO_2, pH 7.4) and kept at room temperature before recordings. They were then transferred to a recording chamber and positioned over the electrode grid of a MED64 probe (8 × 8 microelectrode array, electrode size, 50 × 50 μm, interpolar distance, 150 μm, Alpha MED Sciences Inc., Osaka, Japan) using an Inverted optical microscope (XDS-2, OPTIKA, Italy). During recording, slices were submerged and superfused (2 ml/min) with artificial cerebral spinal fluid (ACSF) using a peristaltic pump and maintained at near-physiological temperatures (32~34 °C) using a temperature control system (ThermoClamp-1, AutoMate Scientific, USA). Five minutes of baseline activity were collected before drug perfusion. Concentration of 10 μM CNO was added into the ACSF perfusion circulation for 4 min, after which it was replaced by normal ACSF. Drug effects were measured by recording trace 5 min before and 10 min after drug perfusion. Action potential firing frequency from LC area were recorded and analyzed using the MED64 system (Alpha MED Sciences Inc.).
